# Foregone benefits of important food crop improvements in Sub-Saharan Africa

**DOI:** 10.1371/journal.pone.0181353

**Published:** 2017-07-27

**Authors:** Justus Wesseler, Richard D. Smart, Jennifer Thomson, David Zilberman

**Affiliations:** 1 Social Science Department, Wageningen University, Wageningen, The Netherlands; 2 TUM School of Life Sciences Weihenstephan, Technische Universität München, Freising, Germany; 3 Department of Molecular and Cell Biology, University of Cape Town, Rondebosch, South Africa; 4 Department of Agricultural & Resource Economics, University of California, Berkeley, California, United States of America; International Nutrition Inc, UNITED STATES

## Abstract

A number of new crops have been developed that address important traits of particular relevance for smallholder farmers in Africa. Scientists, policy makers, and other stakeholders have raised concerns that the approval process for these new crops causes delays that are often scientifically unjustified. This article develops a real option model for the optimal regulation of a risky technology that enhances economic welfare and reduces malnutrition. We consider gradual adoption of the technology and show that delaying approval reduces uncertainty about perceived risks of the technology. Optimal conditions for approval incorporate parameters of the stochastic processes governing the dynamics of risk. The model is applied to three cases of improved crops, which either are, or are expected to be, delayed by the regulatory process. The benefits and costs of the crops are presented in a partial equilibrium that considers changes in adoption over time and the foregone benefits caused by a delay in approval under irreversibility and uncertainty. We derive the equilibrium conditions where the net-benefits of the technology equal the costs that would justify a delay. The sooner information about the safety of the technology arrive, the lower the costs for justifying a delay need to be i.e. it pays more to delay. The costs of a delay can be substantial: e.g. a one year delay in approval of the pod-borer resistant cowpea in Nigeria will cost the country about 33 million USD to 46 million USD and between 100 and 3,000 lives.

## Introduction

“*There is uncertainty and confusion in many of the African governments’ responses to a wide range of social*, *ethical*, *environmental*, *trade and economic issues associated with the development and application of modern genetic engineering*. *The absence of an African consensus and strategic approaches to address these emerging biotechnology issues has allowed different interest groups to exploit uncertainty in policymaking*, *regardless of what may be the objective situation for Africa*.” [1]

A number of new crops have been developed that address important traits of relevance to smallholder farmers in Africa [2]. A sizeable body of literature (see survey by [3]) argues that flows in the regulatory system, partially caused by political economic considerations [4], caused scientifically unjustified delays in the approval process for these new crops. Such delays often result in the bizarre situation where technologies that both increase consumer and producer surplus also have the potential for meaningfully decreasing malnutrition, fail to reach the market. The objective of this paper is to assess the costs caused by those delays under uncertainty and irreversibility.

In this article we investigate three genetically engineered (GE) crops for Africa in more detail, namely disease resistant cooking banana (matoke), and insect resistant cowpea, and corn (maize). A yield increase for those crops can improve the dietary energy supply and have a positive impact on malnutrition [5].

The disease resistant banana [6] and herbicide resistant corn [7] have been available for field trials since the mid- to late 2000s, while the herbicide resistant cowpea has recently received approval for field trials in Nigeria [8]. Although delays for the corn and banana have already been observed, further delays can be expected, including for cowpea.

Despite the clear link between agricultural productivity and malnourishment, many countries in Africa are reluctant to approve GE crops. African governments find themselves juxtaposed between the opponents and proponents of the technology.

Here, we develop a theoretical model assessing the benefits and costs of approval processes using a real option framework calling upon the ‘Santaniello Theorem of Irreversible Benefits’ [9]. The model explicitly considers the standard welfare measures of changes in producer and consumer surplus. Many studies on GE crops have focused on the economic surplus at farm, regional, or sector levels. We contribute to the literature by also considering the effects of GE crops on malnutrition, which is an effect often acknowledged (e.g. [10]), but has received scant attention in the economic literature (notable exceptions: Vitamin A enriched rice [11,12], bio-fortified cassava [13]).

We calculate the foregone benefits caused by a delay in approval under irreversibility and uncertainty, and threshold values that would justify a delay. We consider differences in the approval time of a new crop, and derive the equilibrium conditions (where the net-benefits of the technology equal potential costs) that would justify a delay. We calibrate the model for the three crops considered to indicate the magnitude of the effects, and crucially, the economic and humanitarian consequences of delaying approvals.

The results show that about two thirds of uncertainty is sufficient to compensate for three thirds of certainty. Delays are costly and the effects on malnutrition can sometimes exceed the effects on producer and consumer surplus, and may even be much larger, especially for the case of cowpea (a protein-rich crop) as we only consider the crops’ energy content.

## Approval delays of GE crops in Africa

Bt cotton was the first GE crop approved for cultivation in Africa and was introduced into South Africa in 1997, followed by yellow and white corn in 1998 and 2001, respectively [14]. The first field trials of GE crops in South Africa started in 1989. It took seed companies about nine years to identify and multiply the appropriate corn varieties, a time frame that is usual in plant breeding. If the private sector had approached Kenya or other African countries simultaneously, it is reasonable to expect that local corn varieties with insect- and herbicide resistance would have also been available shortly after the year 2000. In Kenya the first varieties for release were recommended in 1998 [15]. The National Agricultural Research Organisation of Uganda (NARO) submitted applications to the Uganda National Council for Science and Technology (UNCST) in 2000 to introduce Bt cotton and Bt corn, but their approval for confined field trials was denied [16]. One of the reasons the UNCST gave was that Uganda was unprepared to handle GE crops because it lacked a national biotechnology and biosafety policy. The progress of the Insect Resistant Maize for Africa Project (IRMA) was similarly delayed by regulatory issues [17].

In Kenya, under the IRMA (started in 1999) [18] and the Water Efficient Maize for Africa (WEMA, started in 2008) projects for insect and drought resistant corn, varieties are under development with field trials at different stages. Kenya banned the import and cultivation of GE crops in 2012 due to health concerns [19], but is currently considering removing the ban [20]. If the development of this crop under the IRMA project had proceeded as planned and approval not been delayed, the first varieties would have appeared on farmers’ fields in 2006 [18].

In Uganda, field trials with black sigatoka (also known as black leaf streak) resistant matoke (cooking banana) started in 2007 [21]. A bacterial wilt resistant matoke is under development. Field trials have been in place since 2011 [22], and its release to farmers is expected in 2020.

Research in Benin, Niger, and Nigeria (under the coordination of the African Agricultural Technology Foundation (AATF)) to develop cowpea resistant to pod borers started in 2008. Confined field trials commenced in 2010, and it is expected that seeds will be available for farmers by 2017, subject to approval from regulatory agencies [23]. An overview about the regulatory status of the three crops considered is presented in Table 1.

**Table 1:** Status of crops considered.

Country	Benin, Niger, Nigeria[24]	Kenya[17,18]	Uganda[21]
Crop	Cowpea (Vigna unguiculata)	White Corn	Matoke
Trait	Insect resistance	Insect resistance, stress tolerance	Black sigatoka resistance, Bacterial Wilt Resistance
genetic event/ genes introduced	Cry1Ab	Examples: MON810, Event 176, Event 5207	Chitinase gene (Black Sigatoka), hypersensitivity response-assisting protein (Hrap) gene from sweet pepper (bacterial wilt).
Partners Involved	AATF, CSIRO, IAR, IITA, INERA, Monsanto Company, NARS, NGICA, The Kirkhouse Trust	AATF, KALRO (former KARI), CIMMYT, Monsanto Company, University of Ottawa, NARS, Syngenta Foundation, Rockefeller Foundation, USAID	Academia Sinica, NARO, IRAZ, IITA, Public and private tissue culture laboratories in the Great Lakes region of Africa including Burundi, Democratic Republic of Congo, Kenya, Rwanda, Tanzania and Uganda
Regulatory Status	confined field trials since 2011	National Performance Trials (NPT) since 2004	confined field trials since 2007
Expected release^a^	2017^b^	Since 2006^c^	Since 2007^d^ 2020
Country Policy	Cartagena Protocol signed in 2000	Cartagena Protocol signed in 2000 National cultivation and import ban since 2012.	Cartagena Protocol signed in 2000

Sources: references mentioned and project websites: http://aatf-africa.org/.

^a^Expected release refers to reports. As none has been released so far early dates indicate regulator delays.

^b^Expected by 2017 depending on regulatory approval.

^c^According to KARI and CIMMYT, first varieties should have reached farmers field by 2006, while first recommendations for release have been submitted in 1998.

^d^The status of the Black Sigatoka resistant banana is not known. Several experts involved in the research as well as the deregulation had been contacted. For the bacterial wilt resistant banana confined field trials are undertaken and release to farmers is expected for 2020.

Abbreviations: AATF: African Agricultural Technology Foundation, CIMMYT: International Maize and Wheat Improvement Center, CSIRO: Commonwealth Scientific and Industrial Research Organisation, IAR Institute of Agricultural Research, Zaria, Nigeria, IITA: International Institute of Tropical Agriculture, INERA: Institut de l’Environnement et de Recherches Agricoles, Burkina Faso, IRAZ: Institut de recherche agronomique et zootechnique, KALRO: Kenya Agricultural and Livestock Research Organisation, NARO: National Agricultural Research Organisation of Uganda, NGICA: Network for the Genetic Improvement of Cowpea for Africa, NARS: National Agricultural Research Systems in target countries of west Africa.

Further, Benin, Kenya, Niger, Nigeria, and Uganda signed the Cartagena Protocol in 2000 [25]. The interpretation at national level is that they must first have a biosafety law in place before approving GE crops for cultivation. The protocol does, however, provide exemptions under Article 11 in cases where countries have not yet passed a biosafety law [26]. Nevertheless, in Africa, the development of a biosafety law is often used as an instrument in the political process to delay the introduction of GE crops [4].

## Benefits and costs of delay

We are interested in the minimum additional costs that policy makers implicitly perceive would justify postponing the introduction of the crops considered. The model used is explained in detail in S1 File The General Analytical Model: In particular, we assume that the policy makers know with certainty the benefits of the crops in terms of consumer and producer surplus, and malnourishment, but are uncertain about the wider impact. Those uncertainties are modelled as a random shock. Thus, to account for this uncertainty there is a threshold of the benefit from the use of the technology one period earlier that has to be exceeded at each moment in order to approve the technology for use—otherwise the regulator should delay the decision by one or more periods to gain more information. We computed that this threshold of benefits is the expected cost of earlier adoption multiplied by a coefficient that is decreasing as the variability of the cost affecting the random shock is increasing (see eq. 13 in S1 File The General Analytical Model). Our analysis shows that for a delay in approval, the increase in benefit by one dollar under reasonable assumptions requires only an increase in the cost of adoption of about 2/3 of a dollar. Thus, the tendency to overregulate the technology may be explained by the low cost of regulation relative to the benefit of adopting the technology.

The expected economic benefits of cultivating Bt corn [7], Bt cowpea [27], and disease resistant bananas [6] are expected to be substantial. The total surplus reported by studies using partial equilibrium models range from 280 to 360 million USD, 90 to 154 million USD, and 6 to 48 million USD, for bananas, cowpea, and corn, respectively (Table 2). We use this information and apply the linear supply and demand model with a logistic adoption function (see S1 File The General Analytical Model for the details) to calculate the expected average annual consumer and producer surplus. We report the results for a range of supply and demand elasticities commonly found in the literature for these crops [28, 29] (Table C in S1 Table). If not mentioned otherwise, results are reported for short-run own demand and supply elasticity of -0.3 and 0.6, respectively.

**Table 2:** Benefits and costs of GE crops considered.

Crop	Banana	Cowpea	Corn
Country	Uganda[6]	Benin, Niger, Nigeria[27]	Kenya[7]
Traits	disease resistance (black sigatoka, bacterial wilt)	pest resistance (maruca pod borer)	pest resistance (stem borers)
Benefits	reduced damage loss, better quality	reduced damage loss, less mycotoxins	reduced damage loss, less mycotoxins,
Δ Yield/ha	2.0t (20%)	12.5%	0.06–0.3t
Δ Rev/ha	280–450 USD		10–55 USD
Δ PS/a	280–360 Mio. USD	-61–186 Mio. USD	2.0–16.1 Mio. USD
Δ CS/a		-31–77 Mio. USD	4.0–32.2 Mio. USD
Δ TS/a	280–360 Mio. USD	90–154 Mio. USD	6.0–48.3 Mio. USD
K-Shift	0.16 (19.8%)	0.10 (12.5%)	0.11 (13.4%)

Note: results derived from the studies mentioned for each country in the superscript.

### The Country-level cost of stunting

The changes in consumer and producer surplus exclude additional benefits that might arise due to changes in malnutrition. Assessing those benefits requires information about malnutrition and related costs. We measure effects on malnutrition by using changes in stunting, as those are well documented. Stunting reflects a failure of the human body to reach linear growth potential because of suboptimal health and/or nutritional conditions (see S2 File Calculating the Costs of Stunting for the details). Stunting at national level represents the percentage of children below the age of five years with more than minus two standard deviations below the median height-for-age of the World Health Organization (WHO) Child Growth Standards [30].

Table 3 gives an overview of malnutrition in the five countries we consider, and forms part of the data we use for calculating changes in malnutrition. More than ten percent of stunted children worldwide live in these countries. Nigeria has the worst situation with more than 11 million stunted children, followed by Kenya and Uganda. The situation is worse in rural than in urban areas, except in Niger.

**Table 3:** Status of malnourishment in Benin, Niger, and Nigeria, Kenya, and Uganda for the year 2011 [30].

	Benin	Niger	Nigeria	Kenya	Uganda
	Cowpea			Corn	Matoke
Children below six (thousand)	1546	3196	27195	6805	6638
Stunting^a^ in per cent of children below five years of age	43 (<1)	51 (1.0)	41 (6.8)	35 (1.5)	33 (1.4)
Children stunted (thousand)	572	1,585	10,029	1,839	2,318
Children stunted rural areas (thousand)	337	763	5,938	1015	1405
Consumption (kg per head and year of crop)	9[31]^b^	1.5[31]^b^	18[31]^b^	98[32]	300[33]
Consumption increase (kg per year)	2.25	0.375	4.5	14	60
Calories supplied by yield increase per year	2610	435	5220	51100	53400
Per cent of demand ≅ effect on stunting in per cent[34]	0.51	0.09	1.02	10.00	10.48
Current costs of stunting (Mio USD per year)	572	1,585	10,029	1,839	2,318
Current costs of stunting in rural areas (Mio USD per year)	337	763	5,938	1015	1405
Cost reduction (Mio USD/year)	1.72	0.65	60.66	101.54	146.83
Cost reduction (Mio USD/year)^c^[5]	0.48	0.18	16.85	10.53	15.23

Note: Current costs per country estimated by 1000 USD per stunted child.

^a^Number in brackets indicate world share.

^b^Grams per day per household multiplied by 365 and divided by 5 members per household, providing a range between 2.99 and 14.60 kg per year. A value of 10kg per year has been chosen.

^c^Based on estimations by Smith and Haddad.

Calculating the costs related to stunting is not a trivial exercise. The costs include those related to early childhood death and losses in labour productivity. We use the number provided by The World Bank [35] on productivity losses caused by stunting for Africa and Asia of 1,000 USD per child below the age of five years (present value). The details of our calculations are provided in the supplement (Calculating the Costs of Stunting). The current costs of stunting in rural areas (Mio USD per year) are very much on the low side. Other estimations show much higher costs [36].

## Results—The cost of regulatory policy

**Fig 1:**
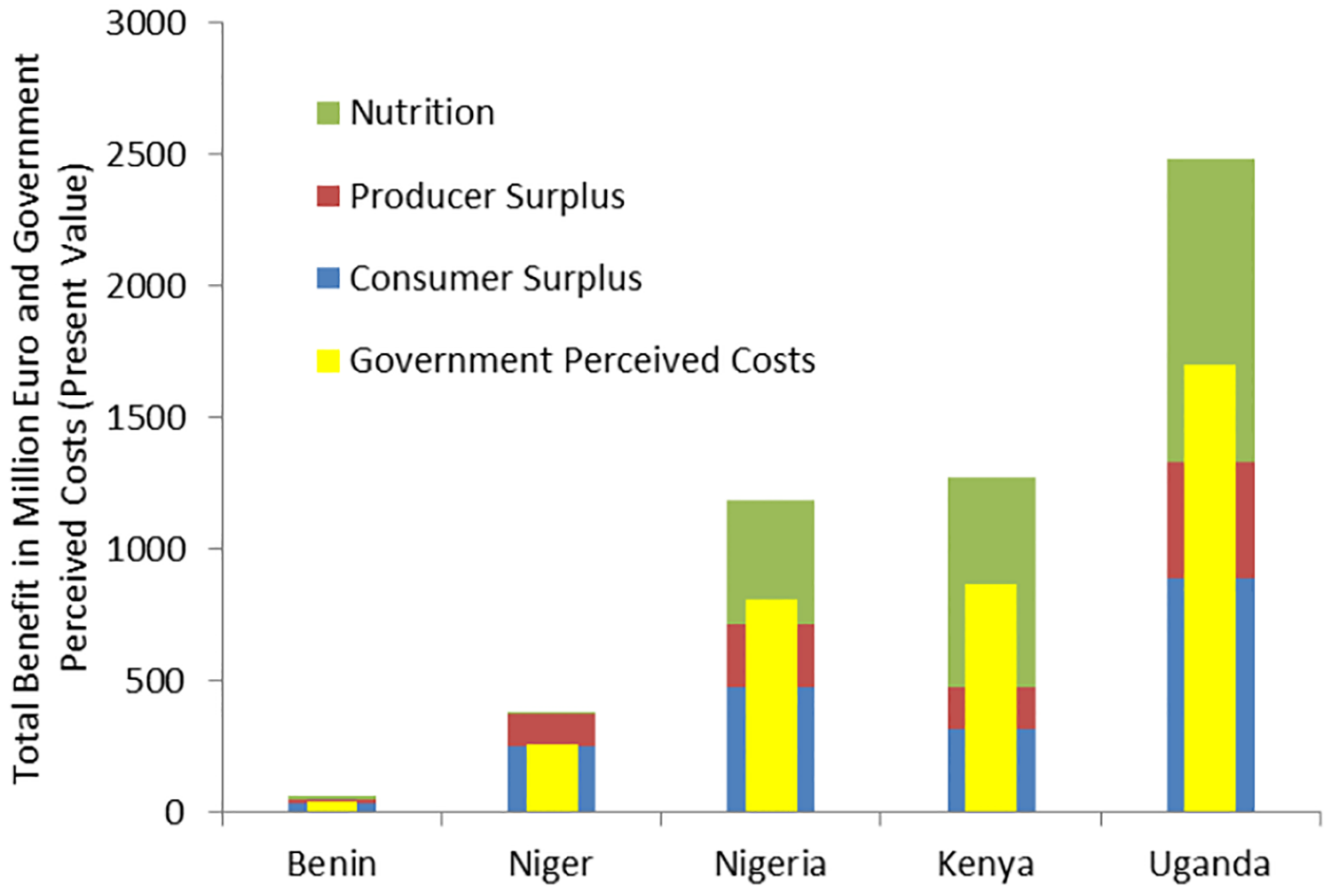
Consumer and producer surplus, benefits of reduced malnutrition, minimum amount of government perceived costs for a one year delay in approval (million USD). Note. Parameter values: adoption ceiling of 40% after 20 years; discount rate r = 0.04; d = 0.5; elasticity of supply *ε = 0*.*6*, elasticity of demand *η = -0*.*3*.

A delay in approval results in welfare losses due to foregone benefits. In our analysis the foregone benefits include: foregone consumer and producer surplus, and foregone reductions in malnutrition (measured as reduced stunting among children). The results shown in Fig 1 and in S1A Table, assume an immediate introduction of the GE crops and report the resultant consumer and producer surplus, plus the benefits of reduced stunting. In general, the consumer surplus is twice as large as the producer surplus for *η* is -0.3 and *ε* is 0.6. The total surplus (net present value) is the largest for GE matoke in Uganda with about 1,300 million USD, followed in descending order by: cowpea in Nigeria with about 710 million USD, corn in Kenya with about 475 million USD, and cowpea in Niger and in Benin with about 375 million USD and about 47 million USD, respectively. The average annual consumer and producer surplus is reported in brackets (Table 4).

**Table 4:** Foregone benefits (consumer and producer surplus, benefits of reduced malnutrition) for a one and ten year delay in approval (million USD).

	Benin		Niger		Nigeria		Kenya		Uganda	
	Cowpea						Corn		Matoke	
	1 year	10 year	1 year	10 year	1 year	10 year	1 year	10 year	1 year	10 year
Foregone: - consumer surplus	1.23	10.33	9.82	82.60	18.65	156.80	12.42	104.46	34.87	293.16
- producer surplus	0.61	5.17	4.91	41.30	9.32	78.40	6.21	52.23	17.43	146.58
- total surplus	1.84	15.50	14.74	123.91	27.97	235.19	18.64	156.69	52.30	439.74
- reduced stunting	0.53	4.44	0.20	1.68	18.61	156.49	31.16	261.96	45.05	378.78
- reduced stunting^SH^[5]	0.15	1.23	0.06	0.47	5.17	43.47	3.23	27.17	4.67	39.28
Total	2.37	19.93	14.94	125.58	46.58	391.68	49.79	418.65	97.35	818.52
Total^SH^	1.99	16.73	14.79	124.37	33.14	278.66	21.87	183.86	56.97	479.02

Note: superscript SH denotes calculation for malnutrition based on Smith and Haddad. Parameter values: adoption ceiling of 40% after 20 years; discount rate r = 0.04; d = 0.5; elasticity of supply *ε = 0*.*6*, elasticity of demand *η = -0*.*3*.

The effect of alleviating malnutrition by using GE crops can be substantial. In Kenya, the benefits from reduced malnutrition can be larger than the total economic surplus. The benefits from reduced malnutrition can be up to about 1,150 million USD for matoke in Uganda, followed by about 795 million USD for corn in Kenya. The effects are also substantial for cowpea in Nigeria with about 475 million USD, while they are smaller for Benin with about 13 million USD and Nigeria with about five million USD. The average annual benefits of reduced stunting reported in Fig 1 are lower than those in Table 3 as adoption over time has been taken into consideration (40 percent ceiling reached after 20 years) for the former.

**Fig 2:**
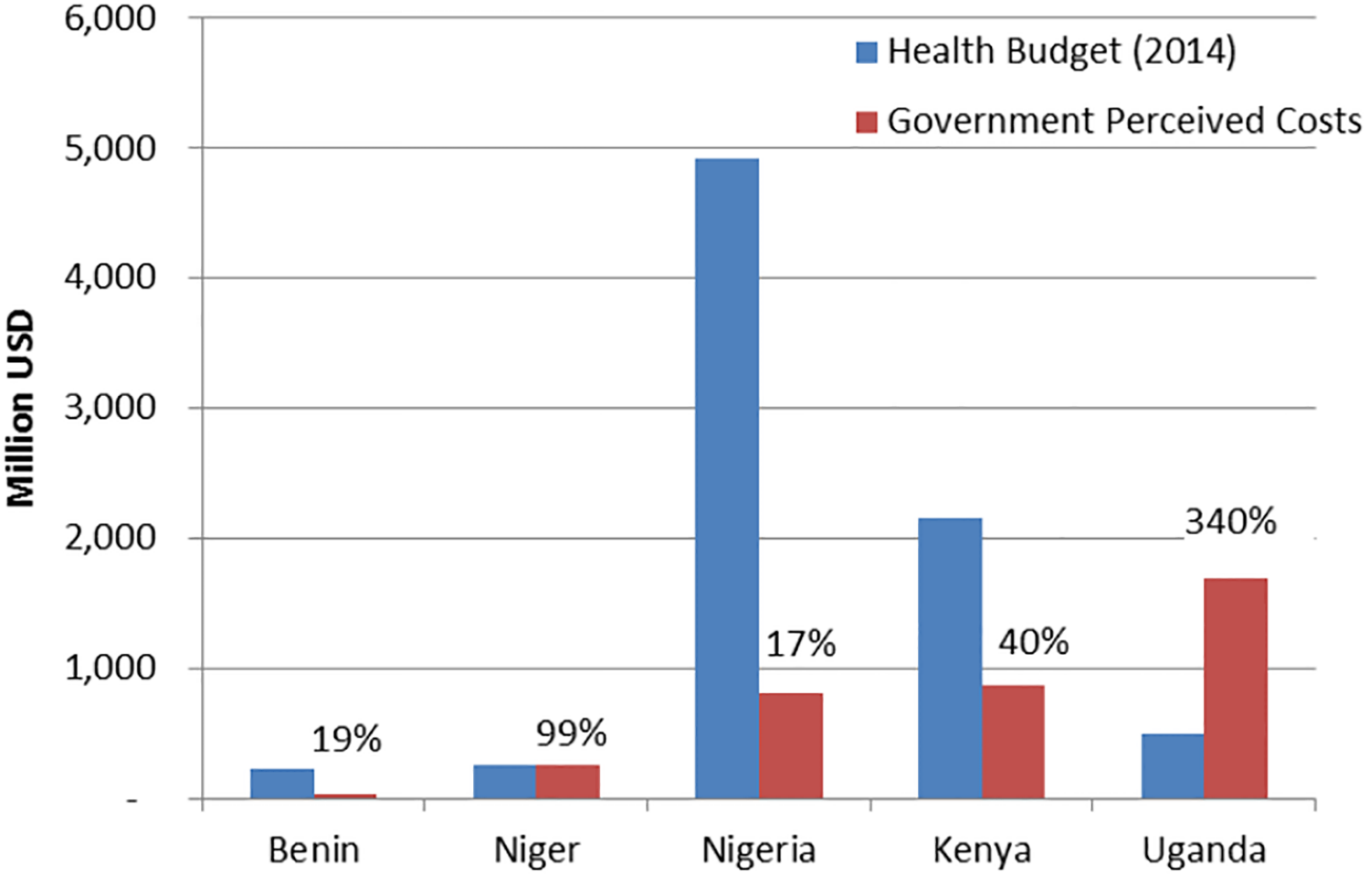
Comparing government perceived costs with health budget of 2014 [37]. Note. Parameter values: adoption ceiling of 40% after 20 years; discount rate r = 0.04; d = 0.5; elasticity of supply *ε = 0*.*6*, elasticity of demand *η = -0*.*3*.

The minimum perceived costs by national governments that would justify a delay from a welfare economic viewpoint are lower than the welfare benefits: They are about 68 percent (based on the following: discount rate of 0.04, value of d = 0.5, demand elasticity of -0.3, supply elasticity of 0.6, and an adoption ceiling of 40 percent reached after 20 years) (see Fig 1). The minimum perceived costs are the lowest for Benin (about 40 to 48 million USD), and the highest for Uganda (about 1,160 to 1,983 million USD). The perceived costs increase with an increase in the time of delay, for e.g., an increase in delay from one to ten years increases the perceived costs, *c*.*p*., by about 17 percent (see S1A Table). Placing these numbers in perspective (Fig 2) shows their importance: They are similar to Niger’s health budget and more than three times Uganda’s health budget.

Table 4 reports the forgone benefits caused by delays in approval, which range between about 2 million USD and 97 million USD, and about 17 million USD and 818 million USD for a one and ten year delay, respectively. As with the share of benefits from reduced malnutrition, the share of foregone benefits of reduced malnutrition can be substantial. In Kenya (similar to the results reported above), these benefits can be larger than the foregone economic surplus.

**Fig 3:**
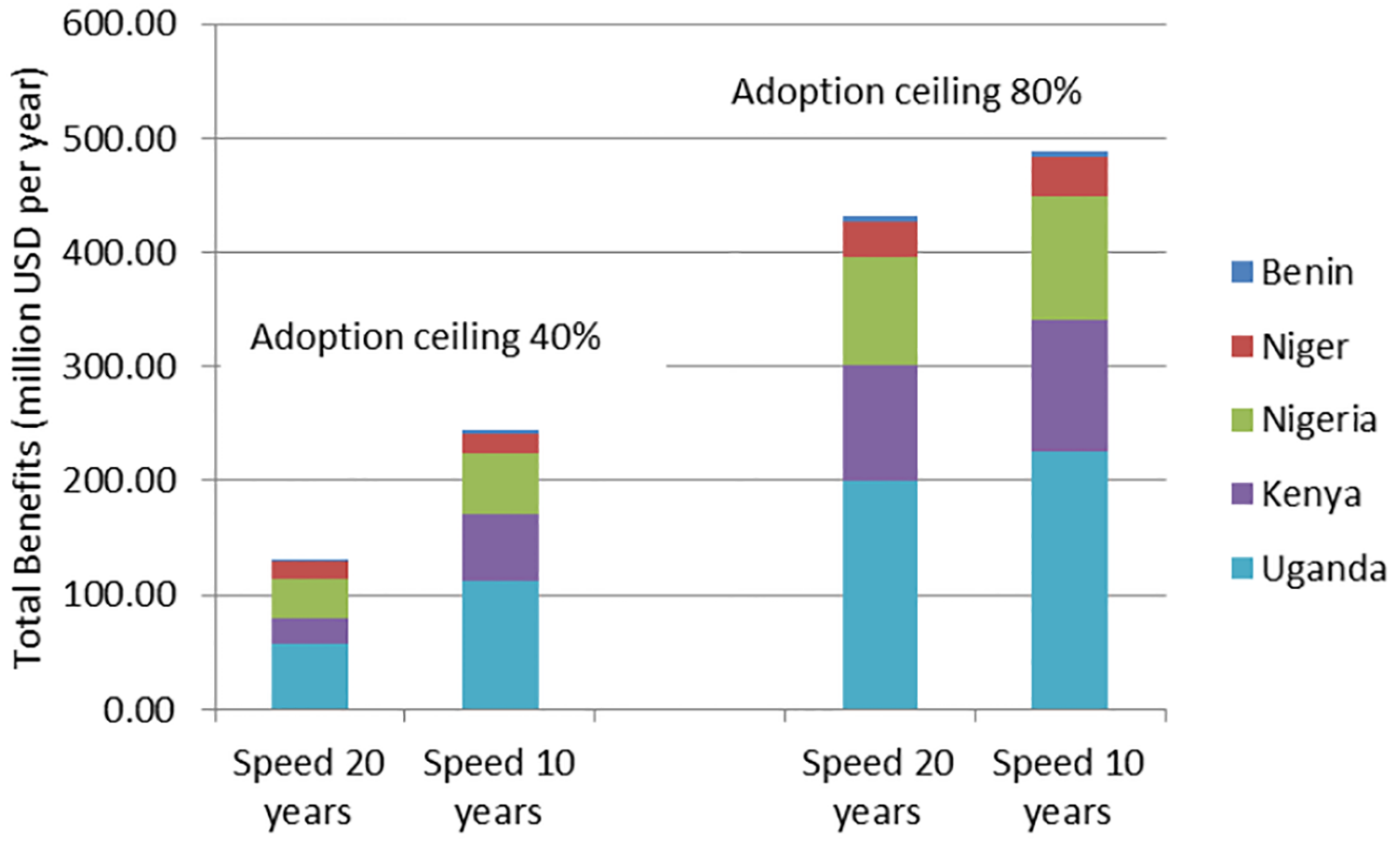
Comparison of doubling the speed of adoption and the ceiling of adoption. Note. Parameter values: discount rate r = 0.04; d = 0.5; elasticity of supply *ε = 0*.*6*, elasticity of demand *η = -0*.*3*. See Table C in S1 Table for different elasticities.

Fig 3 and S1B Table report the results of changes in the adoption ceiling, as well as the speed of adoption expressed in lives lost, which illustrate the effects of the GE crops on food deficient households. We report results for 40, 80, and 100 percent adoption ceilings and two rates of adoption: Ceiling reached after 20 and 10 years, respectively. The number of lives lost by delaying the introduction range between about 200 and 5,500, depending on the speed of adoption, adoption ceiling, and the method used for calculating malnourishment. The results illustrate that a higher adoption ceiling has a much stronger effect on the death toll than a higher adoption rate (Fig 2).

**Fig 4:**
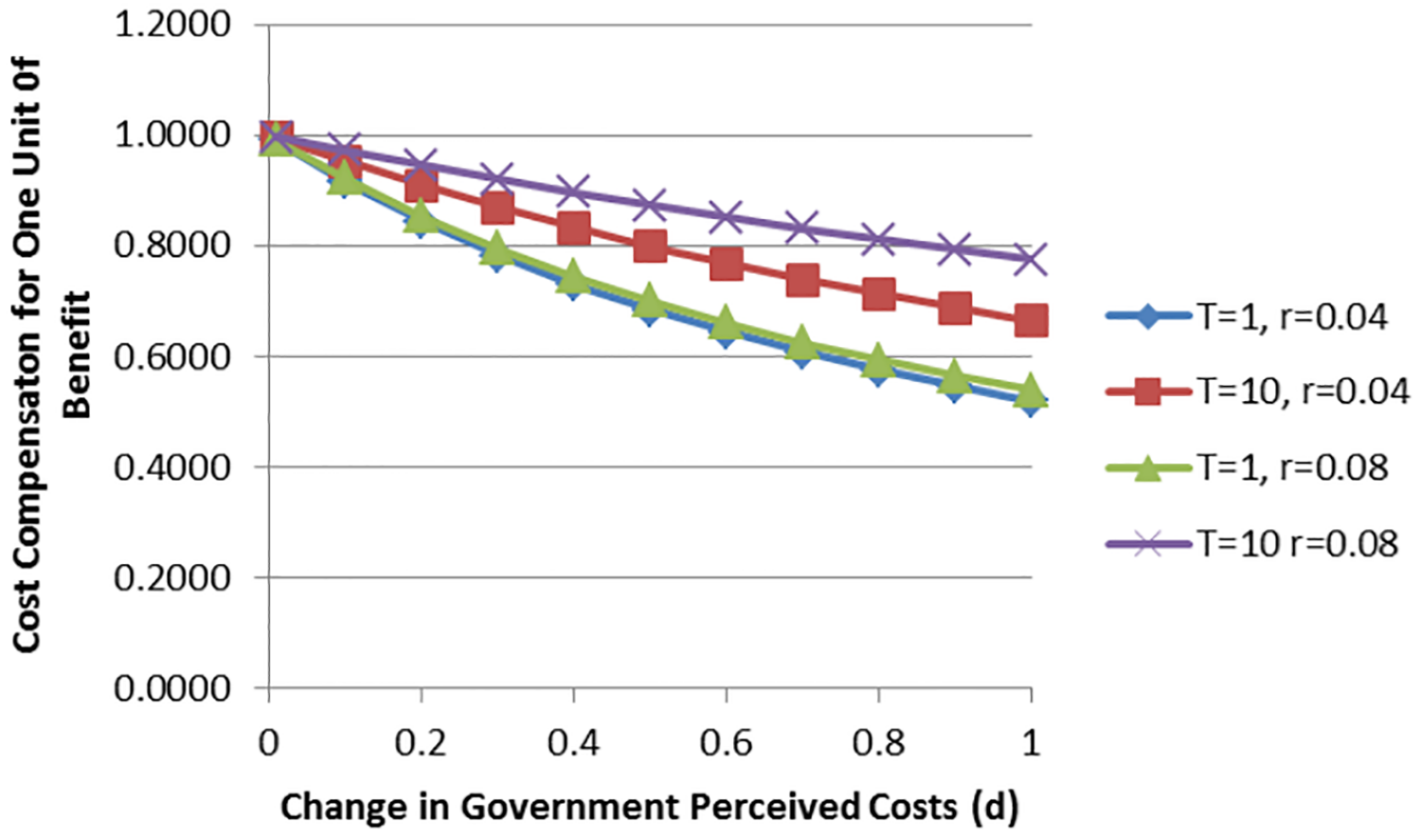
Effect of changes in government perceived costs (d) on cost needed to compensate for one unit of benefit for different discount rates, r, and length in delay, T.

The effect of the length in delay, as well as changes in the discount rate for different levels of *d* on the perceived government costs, are shown in Fig 4. The share of perceived costs needed to compensate one unit of welfare benefits decreases with an increase in *d* at a decreasing rate. The effect of a marginal change in the discount rate is the same as a marginal change in the length of the delay (see equation 13 in S1 File The General Analytical Model). The lower the value of *d*, the larger the effect of marginal changes in *d* (i.e. a relatively high elasticity) on changes in the perceived costs of the government.

The effects of delaying the introduction of a GE crop on foregone benefits also depend on the demand and supply elasticities. The results reported so far were based on a demand elasticity of −0.3 and a supply elasticity of 0.6. Foregone benefits have also been calculated for lower and higher elasticity values (see S1C Table). In general, we observe that a change in the demand elasticity has a less pronounced effect than a change in the supply elasticities. Overall, the effect of changes in the elasticities on foregone benefits are less pronounced than changes in adoption ceiling.

## Discussion and conclusion

For opponents of the technology, announcing uncertainty about the GE crops shortly before decisions are to be made about them can be an effective tool for delaying their introduction. High uncertainty reduces the perceived costs needed (about two thirds) to compensate for one unit of welfare benefits. This uncertainty eases the opponents’ success in delaying approval compared to the proponents’ efforts at speeding up the approval process. This finding also applies to cases not discussed by us and to the approval process for GE crops in general. Delays in approval have been observed for countries outside of Africa too [38, 39]. The results further support the findings by Tosun et al. [40] that show opponents compared with proponents of GE crops time their activities better, have much larger networks, and are more active.

Few studies have investigated the costs of a delay. Most ex-ante studies identify the benefits and costs of the introduction of a new GE crop, which are different to those of a delay. Nevertheless, estimating producer and consumers surplus is an important step. The producer and consumer surplus we report for the three crops are somewhat lower than those reported by others [6, 7, 27]. In comparison to previous studies on the benefits and costs of GE crops, we include their effect on malnutrition, which we show can be substantial. For Kenya, the effect on malnutrition can even be larger than the effect on producer and consumer surplus. This illustrates that in countries where malnutrition is of importance the effect should be considered in the analysis of welfare effects. This further illustrates that the effect on malnutrition of GE crops and other yield increasing strategies deserve attention, so that their economic and humanitarian effects are not underestimated.

Kenya and Uganda (and many other African countries) had the chance to follow South Africa’s example of adopting GE crops. If Kenya had adopted GE corn in 2006—according to the reports of the IRMA project this was possible—between 440 and 4,000 lives could theoretically have been saved. Similarly, Uganda had the possibility in 2007 to introduce the black sigatoka resistant banana, thereby potentially saving between 500 and 5,500 lives over the past decade. The introduction of Bt cowpea is expected to be in 2017 in Benin, Niger, and Nigeria. The AATF has already indirectly expressed concerns about reaching this goal by explicitly mentioning the phrase: “depending on approvals” [41]. A one-year delay in approval would especially harm Nigeria, as malnourishment is widespread there. The consumption of cowpea per capita is higher than in both Benin and Niger. A one-year delay is estimated to cost Nigeria about 33 million USD to 46 million USD and between 100 and 3,000 lives.

Our results might have underestimated the cost of delay, especially in evaluating the benefit of adopting insect resistant cowpea, as we only consider the energy content of this crop. Further, environmental and health benefits from reduced pesticide use for pest and disease control are not explicitly included—an important area for future research.

Nevertheless, our results show that delaying the approval of GE crops not only reduces consumer and producer surplus of households (mainly in rural areas), but importantly, it also costs human lives. We have expressed the effects of a one-year delay on lives lost. The death toll can be substantial. Reducing the approval time of GE crops results in generating economic gains, potentially contributing to reducing malnutrition and saving lives, and can be an inexpensive strategy for reaching the Sustainable Development Goal of eradicating malnutrition by 2030.

Unfortunately, the use of GE crops has been very controversial. African governments are in the dilemma as they face contradicting statements from international organizations. While those organizations (e.g. the UN [42]) stress the importance of addressing malnutrition and urge countries to use modern biotechnology, they also warn about the environmental risks of using the technologies [43]. Unsurprisingly, governments are uncertain about which is the right strategy to follow. We have calculated the economic value of this uncertainty, which is substantial and costs lives. As already mentioned, about two thirds of uncertainty are sufficient to compensate for three thirds of certainty.

## Supporting information

S1 File The General Analytical Model. (DOCX)

S2 File Calculating the costs of stunting. (DOCX)

S1 Table (DOCX)
