# Supplementary material for: Foregone benefits of important food crop improvements in Sub-Saharan Africa
Source: PLoS One. 2017 Jul 27;12(7):e0181353. doi: 10.1371/journal.pone.0181353 (PMC5531496; doi:10.1371/journal.pone.0181353)
Supplement: S1 File — (DOCX) [file pone.0181353.s001.docx]

**Foregone Benefits of Important Food Crop Improvements in Sub-Saharan Africa**

Justus Wesseler^1^, Richard D. Smart^2^, Jennifer Thomson^3^, David Zilberman^4^

* Corresponding Author

E-mail: justus.wesseler@wur.nl

**S1. The General Analytical Model**

**S1. The General Analytical Model**

We develop a modified welfare economic framework for a national government regulating the approval of GE crops. It is a dynamic framework that considers the effects an introduction will have on consumers’ and producers’ surplus, the perceived uncertain negative external social cost (similar to [1]), and importantly, the direct effects on malnutrition. The negative external effects include implications for international trade, social unrest in the country, potential negative impacts on the environment and human health. Our assumptions are a simplification, but can be justified by the studies investigating the political debates of introducing GE crops in developing countries. Many societal groups, e.g., have declared their intention to protest against the introduction of GE crops, and warn about the negative implications for international trade and long-term implications for agriculture sustainability [2, 3, 4]. The public debates following the FAO’s 2004 SOFA [The State of Food and Agriculture] report on “Agricultural Biotechnology: Meeting the Needs of the Poor?” [5], which has been heavily criticized for its “pro GMO [genetically modified organism]” view or similarly, and the 2009 report of the International Assessment of Agricultural Knowledge, Science and Technology for Development (IAASTD) [6], which has been criticized by The World Bank [7] for paying insufficient attention to the possibilities of modern biotechnology to address food security, are examples highlighting the differing views about the impacts of GE crops by highly influential international organizations. Again, the quote from the introduction summarizes the uncertainties these different views generate for policy makers in developing countries.

We denote the introduction of a GE crop as a change in food policy $F\left( t \right)$, $\Delta F\left( t \right)$, from the current food policy, $F_{0}$, to a new food policy, $F_{1}$. At time *t=0* the government’s view is that the perceived costs, $G_{c}$, of introducing the GE crop exist and are high, $G_{c}\gg0$, while other benefits and costs discussed in more detail below are assumed to be known. Hence, all remaining uncertainty is captured under perceived costs. Over time, further information about the perceived costs arrive and at time, *T,* either the strategy will be successful and perceived costs be small, $\underline{G_{c}}$, with probability, (*1-q*), or confirmed to be high, $\overline{G_{c}}$, with probability, q*.* Hence, the introduction mainly depends on the perceived costs of introducing GE crops. Based on this, the national government may decide the strategy will be introduced immediately, (*T=0*), or postponed, (*T>0*), with, *T,* the optimal time to introduce the GE crop.

Considering these uncertainties, the objective of the decision maker can be described as follows:

(1) $\max_{T} E_{o}\int_{o}^{\infty} \left( \Delta{CS}_{t},\Delta{PS}_{t}, {\Delta M}_{t},C_{t},G_{c} \right)dt$,

with $E_{0}$ the expectation operator, $\Delta{CS}_{t}$ the change in consumer surplus, $\Delta{PS}_{t}$ the change in producer surplus, $\Delta M_{t}$ the change in malnutrition, $G_{c}$ defined as follows with a symmetric rise or fall indicating that decision makers a priori are not biased towards benefits nor costs., i.e. the future can either be good or bad:

(2) $E\left[ G_{c} \right]=\left\{ \begin{aligned} \bar{G_{c}}=\left( 1+d \right)G_{c0}, &with probability q=0.5 \\ \underline{G_{c}}=\left( 1-d \right)G_{c0}, &with probability \left( 1-q \right)=0.5 \end{aligned} \right.$

and the current value of $G_{c0}≙\left( 0.5(1+d)G_{c0}+0.5(1-d)G_{c0} \right)$.

The annual change in producer and consumer surplus can be derived from a partial equilibrium mode. If we assume linear supply and demand functions we get (see e.g. [8], p. 211):

(4) $\Delta CS=\int_{0}^{\infty} \left( P_{T}Q_{T}Z_{t}\left( 1+0.5Z_{t} \right) \right)e^{-rt}dt$,

(5) $\Delta PS=\int_{0}^{\infty} \left( P_{T}Q_{T}(K_{t}-Z_{t})\left( 1+0.5Z_{t} \right) \right)e^{-rt}dt$,

where $Z_{t}={K_{t}\varepsilon}/\left( \varepsilon+ \right)$, $K_{t}=\left[ \frac{\Delta y}{\varepsilon}-\frac{\Delta VC}{1+\Delta y} \right]a_{t}$, *ε* the supply elasticity, *η* the absolute value of the own-price elasticity of demand, *P* the product price and *Q* the product quantity at time *T* of introduction of food policy *F_1_*, *Δy* the per cent yield increase of the GE crop, and *ΔVC* the relative change in variable costs. Both $\Delta CS$ and $\Delta PS$ can be converted into average annual surpluses by multiplying both by *r* and will be denoted by ${CS}_{a}$ and ${PS}_{a}$ respectively.^[[1]](#footnote-1)^

**Measuring Changes in Malnutrition**

We define malnutrition, *M_t_*, as a state variable, which captures the many dimensions of hunger [9]. Malnutrition is controlled by food policies, *F(t),* that affect food deficiency and are translated by the factor $\beta>0$ to malnutrition, i.e. the higher the level of food deficiency at time *t* the higher the level of malnutrition. There is also an exogenous decline in malnutrition by other factors not directly related to the quantity and quality of food supply, which include improvements in childcare and feeding practices and household environment, and health’s services and are a result of basic courses such as the socio-economic environment [9].

The change in malnutrition is $dM=M\left( F_{t} \right)dt$ with $dM\left( F_{o} \right)=0$ and $dM\left( F_{1} \right)=M_{t}dt$. The annual level of malnutrition reduction benefits, *M_t_,* is measured as the number of stunted individuals in a population younger than five years old—a common measure of malnourishment [9] in rural areas—multiplied by the percentage change in calorie intake, *c,* by the GE crop measured, where the percentage change in crop consumption is the same as the change in yield, valued by the average annual costs of stunting, *m*:

(6) $mM_{t}=mn_{t}c$

with $n=fa\left( t \right)$, where *f* is the fraction of stunted children reached. The total benefits in reduction of malnourishment is $M_{0}=\int_{0}^{\infty} \left( mn_{t}c \right)e^{-rt}$ and dividing this through by *r* provides the average annual benefits $M_{a}={M_{0}}/r$.

*Food Policy Change*

The new policy allows producer and consumer surplus to change, and malnutrition to reduce with the new technology to $F_{1}$, introduced at $T$, hence $F_{t}=F_{0}\left| t<T \right.$ and $F_{t}=F_{1}\left| t\geq T \right.$. At time, *T,* introducing the new technology only pays if $G_{cT}=\underline{G_{c}}$ and not otherwise, also implying that it does not necessarily pay to introduce *F_1_* immediately at *t = 0*. Further, there is no future uncertainty after *T*, i.e. whatever $G_{cT}$ will be, and it will remain at that level until infinity. Three cases can now be assessed.

First, the technology will never be adopted. The value of the decision:

(7) $D^{N}=0$.

Second, the technology will be immediately adopted, i.e. $F=F_{1}$:

(8) $D^{0}\left( F_{1} \right)=\int_{0}^{\infty} \left( {CS}_{a}+{PS}_{a}+M_{a} \right)e^{-rt}dt-G_{c0}$

$=\frac{{CS}_{a}+{PS}_{a}+M_{a}}{r}-G_{c0}$.

Applying standard cost-benefit-analysis, adopting the policy would be economical if:

(9) $D^{0}-D^{N}=\frac{{CS}_{a}+{PS}_{a}+M_{a}}{r}-G_{c0}>0$.

Third, the technology will be introduced at time $T$, where the government will know whether or not the social costs will be high, $G_{cT}=\bar{G_{c}}$ or low $G_{cT}=\underline{G_{c}}$. If the social costs are high, the introduction of GE crops will not be useful and their value is zero. If the social costs are low, it would be beneficial from the government’s perspective to introduce them. The value of introducing the GE crop at time, *T,* considering $\left( 1-q \right)=0.5$ (see Eq. 2), from today’s perspective is.

(10) $D^{T}\left( \overline{G_{c}},\underline{G_{c}},F_{1} \right)=\frac{1}{2}\frac{{CS}_{a}+{PS}_{a}+M_{a}}{r}e^{-rT}-\frac{1}{2}\underline{G_{C}}$

The results can now be used to identify whether or not it pays to wait by calculating the difference between postponed and immediate introduction:

(11) $\Delta D^{T}=D^{T}\left( \overline{G_{c}},\underline{G_{c}},F_{1} \right)-D^{0}\left( F_{1} \right)$

$$=\frac{1}{2}\frac{{CS}_{a}+{PS}_{a}+M_{a}}{r}e^{-rT}-\frac{1}{2}\underline{G_{C}}-\frac{{CS}_{a}+{PS}_{a}+M_{a}}{r}+G_{c0}$$

$=G_{c0}-\frac{1}{2}\underline{G_{C}}+\left( {\frac{1}{2}e}^{-rT}-1 \right)\frac{{CS}_{a}+{PS}_{a}+M_{a}}{r}$.

Substituting $\underline{G_{C}}$ with $\left( 1-d \right)G_{c0}e^{-rT}$ provides:

(12) $\Delta D^{T}=G_{c0}\left( \frac{2e^{rT}-1+d}{2e^{rT}} \right)+\left( {\frac{1}{2}e}^{-rT}-1 \right)\frac{{CS}_{a}+{PS}_{a}+M_{a}}{r}$.

Equation 12 can now be used to identify the threshold level of the government’s costs that would result in $\Delta D^{T}>0$, provding:

(13) $G_{c0}>\frac{2e^{rT}-1}{{2e}^{rT}+d-1}\frac{\left( {CS}_{a}+{PS}_{a}+M_{a} \right)}{r}$.

The second term on the right-hand-side (RHS) is the net-present-value (NPV) of consumer and producer surplus plus the benefits from reducing malnutrition. The first term on the RHS shows that the perceived costs by the government have to be lower by the factor $\frac{2e^{rT}-1}{2e^{rT}+d-1}<1$ for each unit of NPV. Using values of *r = 0.04, d = 0.5*, and *T =1* provides a value of 0.68. Hence, government’s perceived costs have to be only 68 per cent of the NPV. Note that care needs to be taken with changes in the discount rate as this affects the first and the second term on the RHS of equation 15.

Equation 13 is a result well-known from the literature on decision making under uncertainty and irreversibility [10, 11]. While most studies develop models considering uncertainty over future reversible benefits and costs, the simple model we developed considers uncertainty over irreversible costs. The model can be advanced by adding an additional cost function (e.g. [12])—this is of less relevance in the context of the problem we are interested in as the crops would be introduced as part of existing dissemination strategies by either the public- or the private sector. Further, adding additional sophistication to the model easily results in problems that cannot be solved analytically, where the mathematical techniques for finding appropriate solutions are currently inadequately developed, thereby easily resulting in problems that are difficult to solve [13].

Now we have a model to identify under what conditions it would be sensible from an economic perspective to either immediately approve or postpone the introduction of a new GE crop, i.e. wait until uncertainty has been resolved and introduce, if $G_{c}$ is low.

The argument here is that by delaying the approval by one year, *T=1*, decision makers assess that the costs exceed the benefits. We identify the marginal costs justifying a delay $\Delta D^{T}=0$ by adjusting $G_{c}$. This results in the following proposition:

*Proposition 1:*

*An increase in uncertainty over the policy costs of the introduction of a GE crop,* ceteris paribus *(c.p.), increases the likelihood that the policy will be introduced later rather than earlier.*

Proof: from equation 12 it can easily be seen that $d\Delta D^{T}\left( \underline{G_{C}},\bar{G_{c}} \right)=\frac{\partial\Delta D^{T}}{\partial\underline{G_{c}}}d\underline{G_{c}}+\frac{\partial\Delta D^{T}}{\partial\bar{G_{c}}}d\bar{G_{c}}<0$.

*Corollary 1:*

*Activities resulting in an increase in uncertainty over the costs of a policy on the introduction of a GE crop, c.p., increases the likelihood that the policy will be introduced later rather than earlier.*

*Corollary 2:*

*Groups opposing the policy have, c.p., an interest in increasing the uncertainty about the net-benefits of the policy.*

In the following we assess the implications of Proposition 1 and the Corollaries for the three crops we are interested in, namely: disease resistant banana, and the cowpea and corn lines that are insect resistant. In the next section we describe the status of these crops within the approval process followed by an assessment of their contributions to reducing: malnutrition, and consumer and producer surplus.

**References**

[1] Wesseler J, Zilberman D. The Economic Power of the Golden Rice Opposition. Environ Dev Econ. 2014;19: 724-742.

[2] Rausser G, Zilberman D, Khan G. An Alternative Paradigm for Food Production, Distribution, and Consumption: A Noneconomist’s Perspective. Annu Rev Resour Economics. 2015;7: 309–31.

[3] Herring R. State science, risk and agricultural biotechnology: Bt cotton to Bt Brinjal in India. J Peasant Stud. 2015;42 (1): 159–186.

[4] Paarlberg R. Starved for Science: How Biotechnology is Being Kept Out of Africa. Cambridge: Harvard University Press; 2008.

[5] FAO. Agricultural Biotechnology: Meeting the Needs of the Poor? Rome: FAO; 2004.

[6] McIntyre RD, Herren HR, Wakhungu J, Watson RT. IAASTD International Assessment of Agricultural Knowledge, Science and Technology for Development: Global Report. Washington DC: Island Press; 2009.

[7] The World Bank. International Assessment of Agricultural Knowledge, Science and Technology for Development (IAASTD). Corporate and Global Evaluations and Methods. Washington DC: Independent Evaluation Group, The World Bank Group; 2010.

[8] Alston JM, Norton GW, Pardey PG. Science under Scarcity. Wallingford UK: CAB International; 1998.

[9] UNICEF. Improving Child Nutrition. New York: UNICEF; 2013.

[10] Arrow KJ, Fisher AC. Environmental Preservation, Uncertainty, and Irreversibility. Q J Econ. 1974;88: 312-319.

[11] Dixit A, Pindyck R. Investment under Uncertainty. Princeton: Princeton University Press; 1994.

[12] Pindyck R. Resource and Energy Economics. 2011;33:761–768.

[13] Balikcioglu M, Fackler PL, Pindyck RS. Solving optimal timing problems in environmental economics. Resource and Energy Economics. 2011;33:761–768.

1. Note: for the spreadsheet model we simplified this by calculating $K_{t}=\left( \Delta y\epsilon\right)a_{t}$. This allows to calculate the changes in variable costs as a residual and to link changes in the supply elasticity to changes in variable costs. [↑](#footnote-ref-1)
