# Supplementary material for: Foregone benefits of important food crop improvements in Sub-Saharan Africa
Source: PLoS One. 2017 Jul 27;12(7):e0181353. doi: 10.1371/journal.pone.0181353 (PMC5531496; doi:10.1371/journal.pone.0181353)
Supplement: S2 File — (DOCX) [file pone.0181353.s002.docx]

**Foregone Benefits of Important Food Crop Improvements in Sub-Saharan Africa**

Justus Wesseler^1^, Richard D. Smart^2^, Jennifer Thomson^3^, David Zilberman^4^

* Corresponding Author

E-mail: justus.wesseler@wur.nl

**S2. Calculating the costs of stunting**

**S2. Calculating the costs of stunting.**

The first step is to compute the contribution of crop improvements to reducing stunting. This is done by adding the percentage increase in yield (by assuming a linear relationship between yield increase and consumption increase at household level) to the average annual consumption in kg per head and country of the respective crops. The average annual consumption per head of matoke bananas in Uganda is about 300 kg [1], while that of corn is about 98 kg in Kenya [2], while that of cowpea covers a wide range from a low of 1.5 kg in Niger followed by 9 kg in Benin to a high of 18 kg in Nigeria [3]. These data are used to compute the increase in consumption per head and year, which range for cowpea from 0.38 kg in Niger, 2.25 kg in Benin, and 4.50 kg in Nigeria to 98 kg for corn in Kenya, and 60 kg for matoke in Uganda.

The second step involves multiplying the number of calories per kg by the average increase in kg per head, which provides the additional amount of calories per year added ranging from a low of 435 calories for Niger to a high of 53,400 calories for Uganda. The percentage of those additional calories on average calorie demand by children below five years of age and year of 511,000 calories is used as the indicator of the percentage reduction in stunting. The annual proportional effect on stunting in ascending order is as follows: 0.09, 0.51, 1.02, 10.00, and 10.48 per cent for Niger, Benin, Nigeria, Kenya, and Uganda, respectively.

We weigh the percentage reduction in stunting by the share of rural stunted population on total population by country. The current costs of stunting using a value of 1000 USD per year and child stunted [4] are about 572 million USD for Benin and up to 10,029 million USD for Nigeria, where in the rural areas it ranges between 337 million USD and 5,938 million USD. The annual reduction in the costs of stunting, assuming full adoption, using the increase in calorie supplies mentioned earlier are about 0.54, 0.20, 18.99, 57.55, and 45.96 million USD for Benin, Niger, Nigeria, Kenya, and Uganda, respectively.

The procedure for calculating the costs of stunting may create biases as the contribution from banana and corn might be overestimated, as calories are but one component of a healthy diet. Further, cowpea is poor in calories, but rich in protein, thus contributing to increasing dietary diversity. As an alternative to assess the contribution to reduce stunting, we use the results provided by Smith and Haddad [5], who estimate the strength of six underlying determinants on child stunting per capita dietary energy supply from what they call staple and non-staple foods using unbalanced panel data. They find that on average an increase of 135 calories from staple foods and a 3.5 per cent increase in calories from non-staple foods would reduce stunting by one per cent. Using these results the effects of an increase in the supply of cowpea was considered as a non-staple food, and an increase in the supply of banana and corn was valued as a staple food (see table 3, last row). Based on this approach the contribution to reduce stunting is lower by a factor ten for banana and corn, and a factor of about four for cowpea. Hence, the relative importance of cowpea has increased. Based on this approach, Nigeria would gain the most followed by Uganda and Kenya. The substantially lower effect on stunting can partially be explained by the fact that the results by Smith and Haddad (SH) [5] were based on the total population, while our approach only considers children, which require a substantially lower (about a half) relative energy intake. Further, SH consider changes in total food supply to be distributed among malnourished and other persons. Finally, the estimations are based on more than a hundred countries and are reported as averages. The five countries we consider have an above average share of malnourished children—covering more than ten per cent of the world’s total number of malnourished children. Hence, using SH’s results is somewhat of a worst-case-scenario.

**References**

[1] Englberger L, Darnton-Hill I, Coyne T, Fitzgerald MH, Marks GC. Carotenoid-rich bananas: A potential food source for alleviating vitamin A deficiency. Food Nutr Bull. 2003;24(4): 303–318.

[2] ACDI/VOCA. Kenya – Kenya Maize Development Program (KMDP). Available from: http://acdivoca.org/our-programs/project-profiles/kenya-kenya-maize-development-program-kmdp. Cited 2 May 2016.

[3] Gómez C. Cowpea: Post-Harvest Operations. Rome: FAO; 2004.

[4] Shekar M. The Costs of Stunting. Washington DC: The World Bank; 2013.

[5] Smith LC, Haddad L. Reducing Child Undernutrition: Past Drivers and Priorities for the Post-MDG Era. World Dev. 2015;68: 180-204.
