# Supplementary material for: Foregone benefits of important food crop improvements in Sub-Saharan Africa
Source: PLoS One. 2017 Jul 27;12(7):e0181353. doi: 10.1371/journal.pone.0181353 (PMC5531496; doi:10.1371/journal.pone.0181353)
Supplement: S1 Table — (DOCX) [file pone.0181353.s003.docx]

**Foregone Benefits of Important Food Crop Improvements in Sub-Saharan Africa**

Justus Wesseler^1^, Richard D. Smart^2^, Jennifer Thomson^3^, David Zilberman^4^

* Corresponding Author

E-mail: justus.wesseler@wur.nl

**S3 Table. Scenario Results.**

**Table A.** Consumer and Producer Surplus, Benefits of Reduced Malnutrition, Minimum Amount of Government Perceived Costs for a One- and Ten-Year Delay in Approval (million USD).

**Table B.** Costs of Stunting of One-Year Delay in Relation to Adoption Ceilings and Speed of Adoption.

**Table C.** Costs of a One-Year Delay in Million USD Per Year for Different Elasticities.

**Table A. Consumer and producer surplus, benefits of reduced malnutrition, minimum amount of government perceived costs for a one- and ten-year delay in approval (million USD).**

|  | Benin | | Niger | | Nigeria | | Kenya | | Uganda | |
| --- | --- | --- | --- | --- | --- | --- | --- | --- | --- | --- |
|  | Cowpea | | | | | | Corn | | Matoke | |
| Benefits: - consumer surplus | 31.33 (1.25) | | 250.56 (10.02) | | 475.60 (19.02) | | 316.86 (12.67) | | 889.23 (35.57) | |
| - producer surplus | 15.67 (0.63) | | 125.28 (5.01) | | 237.80 (9.51) | | 158.43 (6.34) | | 444.61 (17.78) | |
| - total surplus | 47.00 (1.88) | | 375.84 (15.03) | | 713.40 (28.54) | | 475.29 (19.01) | | 1333.84 (53.35) | |
| - reduced stunting | 13.46 (0.54) | | 5.08 (0.20) | | 474.66 (18.99) | | 794.58 (31.78) | | 1148.94 (45.96) | |
| - reduced stunting^SH^ | 3.74 (0.15) | | 1.41 (0.06) | | 131.85 (5.27) | | 82.40 (3.30) | | 119.15 (4.77) | |
| Total | 60.46 (2.42) | | 380.92(15.24) | | 1188.06 (47.52) | | 1269.87 (50.79) | | 2482.78 (99.31) | |
| Total^SH^ | 50.74 (2.03) | | 377.25 (15.09) | | 845.25 (33.81) | | 557.69 (22.31) | | 1452.99 (58.12) | |
| Perceived Government Costs (one and ten year delay) | 41.34 | 48.29 | 260.50 | 304.23 | 812.48 | 948.89 | 868.43 | 1014.23 | 1697.89 | 1982.95 |
| Government Perceived Costs (one and ten year delay) ^SH^ | 34.70 | 40.52 | 257.99 | 301.30 | 578.04 | 675.09 | 381.39 | 445.42 | 993.65 | 1160.48 |

Numbers in brackets show average annual values. Superscript SH denotes calculation for malnutrition based on Smith and Haddad [1]. Parameter values: adoption ceiling of 40% after 20 years; discount rate r=0.04; d=0.5; elasticity of supply *ε=0.6*, elasticity of demand *η=-0.3*.

**Table B. Costs of stunting of one-year delay in relation to adoption ceilings and speed of adoption.**

|  | Benin | | | | Niger | | | | Nigeria | | | Kenya | | | | | Uganda | | | | Total | | | | | | |
| --- | --- | --- | --- | --- | --- | --- | --- | --- | --- | --- | --- | --- | --- | --- | --- | --- | --- | --- | --- | --- | --- | --- | --- | --- | --- | --- | --- |
|  | Cowpea | | | | | | | | | | | Corn | | | | | Matoke | | | |  |  |  |  |  |  |  |
| Adoption Ceiling (%) | 40 | 80 | 100 |  | 40 | 80 | 100 |  | 40 | 80 | 100 |  | 40 | 80 | 100 | |  | 40 | 80 | 100 |  | 40 | | 80 | 100 | | |
|  | Adoption Ceiling after 20 years (one-year delay) | | | | | | | | | | | | | | | | | | | | | | | | | | |
| Lives lost | 9 | 18 | 22 |  | 3 | 7 | 9 |  | 355 | 709 | 886 |  | 505 | 1010 | | 1262 |  | 761 | 1522 | 1902 |  | 1633 | 3266 | | | 4082 | |
| Lives lost^SH^ | 2 | 5 | 6 |  | 1 | 2 | 2 |  | 98 | 197 | 246 |  | 52 | 105 | | 131 |  | 79 | 158 | 197 |  | 233 | 466 | | | 583 | |
|  | Adoption Ceiling after 10 years (one-year delay) | | | | | | | | | | | | | | | | | | | | | | | | | | |
| Lives lost | 10 | 20 | 25 |  | 4 | 8 | 10 |  | 401 | 803 | 1003 |  | 572 | 1143 | | 1429 |  | 862 | 1723 | 2154 |  | 1849 | 3697 | | | 4621 | |
| Lives lost^SH^ | 3 | 6 | 7 |  | 1 | 2 | 3 |  | 111 | 223 | 279 |  | 59 | 119 | | 148 |  | 89 | 179 | 223 |  | 264 | 528 | | | 660 | |
|  | Adoption Ceiling after 20 years (ten-year delay) | | | | | | | | | | | | | | | | | | | | | | | | | | |
| Lives lost | 75 | 150 | 187 |  | 29 | 57 | 72 |  | 2981 | 5961 | 7452 |  | 4246 | 8491 | 10614 | |  | 6398 | 12767 | 15996 |  | 13728 | 27456 | | | 34320 | |
| Lives lost^SH^ | 21 | 42 | 52 |  | 8 | 16 | 20 |  | 828 | 1656 | 2010 |  | 440 | 881 | 1101 | |  | 664 | 1327 | 1659 |  | 1961 | 3921 | | | 4901 | |
|  | Adoption Ceiling after 10 years (ten-year delay) | | | | | | | | | | | | | | | | | | | | | | | | | | |
| Lives lost | 85 | 169 | 212 |  | 32 | 65 | 81 |  | 3375 | 6749 | 8437 |  | 4807 | 9614 | 12017 | |  | 7244 | 14488 | 18110 |  | 15543 | | 31085 | | | 38857 |
| Lives lost^SH^ | 24 | 47 | 59 |  | 9 | 18 | 23 |  | 937 | 1875 | 2344 |  | 498 | 997 | 1246 | |  | 751 | 1502 | 1878 |  | 2220 | | 4439 | | | 5549 |

Superscript SH denotes calculation for malnutrition based on Smith and Haddad (2015). Parameter values: discount rate r=0.04; d=0.5. Lives lost calculated by dividing reduced stunting costs by the life-expectancy per country in years (Benin: 59.3; Niger: 58.4; Nigeria: 52.5; Kenia: 61.7; Uganda: 59.2 [2]) times 1000 USD for the value of a disability-adjusted-life-year (DAILY).

**Table C. Costs of a one-year delay in million USD per year for different elasticities.**

|  | Benin | | | Niger | | | Nigeria | | | Kenya | | | Uganda | | |
| --- | --- | --- | --- | --- | --- | --- | --- | --- | --- | --- | --- | --- | --- | --- | --- |
|  | Cowpea | | | | | | | | | Corn | | | Matoke | | |
| Demand elasticity  Supply elasticity | -0.1  0.4 | -0.3  0.6 | -0.5  0.8 | -0.1  0.4 | -0.3  0.6 | -0.5  0.8 | -0.1  0.4 | -0.3  0.6 | -0.5  0.8 | -0.1  0.4 | -0.3  0.6 | -0.5  0.8 | -0.1  0.4 | -0.3  0.6 | -0.5  0.8 |
| Benefits: - consumer surplus | 0.98 | 1.23 | 1.52 | 7.84 | 9.82 | 12.13 | 14.89 | 18.65 | 23.02 | 9.92 | 12.42 | 15.34 | 27.80 | 34.87 | 43.11 |
| - producer surplus | 0.25 | 0.61 | 0.95 | 1.96 | 4.91 | 7.58 | 3.72 | 9.32 | 14.39 | 2.48 | 6.21 | 9.59 | 6.95 | 17.43 | 26.95 |
| - total surplus | 1.23 | 1.84 | 2.46 | 9.80 | 14.74 | 19.71 | 18.61 | 27.97 | 37.41 | 12.40 | 18.64 | 24.93 | 34.75 | 52.30 | 70.06 |
| - reduced stunting | 0.53 | 0.53 | 0.53 | 0.20 | 0.20 | 0.20 | 18.61 | 18.61 | 18.61 | 31.16 | 31.16 | 31.16 | 45.05 | 45.05 | 45.05 |
| - reduced stunting^SH^ | 0.15 | 0.15 | 0.15 | 0.06 | 0.06 | 0.06 | 5.17 | 5.17 | 5.17 | 3.23 | 3.23 | 3.23 | 4.67 | 4.67 | 4.67 |
| Total | 1.75 | 2.37 | 2.99 | 10.00 | 14.94 | 19.91 | 37.22 | 46.58 | 56.02 | 43.55 | 49.79 | 56.08 | 79.80 | 97.35 | 115.11 |
| Total^SH^ | 1.37 | 1.99 | 2.61 | 9.86 | 14.79 | 19.76 | 23.78 | 33.14 | 42.58 | 15.63 | 21.87 | 28.16 | 39.42 | 56.97 | 74.73 |
| Government Perceived Costs | 30.59 | 41.34 | 52.19 | 174.47 | 260.50 | 347.19 | 649.18 | 812.48 | 977.04 | 759.60 | 868.43 | 978.15 | 1391.84 | 1697.89 | 2007.67 |
| Government Perceived Costs^SH^ | 23.94 | 34.70 | 45.54 | 171.96 | 257.99 | 344.68 | 414.74 | 578.04 | 742.60 | 272.56 | 381.39 | 491.11 | 687.60 | 993.65 | 1303.43 |

Calculations based on a discount rate of *r= 0.04*, a change in perceived costs of *d=0.5*; and a delay of *T=1* year. Superscript SH denotes calculation for malnutrition based on Smith and Haddad [1].

**References**

[1] Smith LC, Haddad L. Reducing Child Undernutrition: Past Drivers and Priorities for the Post-MDG Era. World Dev. 2015;68: 180-204.

[2] The World Bank. Life expectancy at birth, total (years). Available at <http://data.worldbank.org/indicator/SP.DYN.LE00.IN>; 2017.
